# Composite Hydrogels Based on Poly(Ethylene Glycol) and Cellulose Macromonomers as Fortified Materials for Environmental Cleanup and Clean Water Safeguarding

**DOI:** 10.3390/ijms24087558

**Published:** 2023-04-20

**Authors:** Dariya Getya, Alec Lucas, Ivan Gitsov

**Affiliations:** 1 Department of Chemistry, State University of New York—ESF, Syracuse, NY 132101, USA; dgetya@syr.edu; 2 The Michael M. Szwarc Polymer Research Institute, Syracuse, NY 13210, USA; 3 Department of Materials Science and Engineering, Purdue University, West Lafayette, IN 47907, USA; lucas120@purdue.edu; 4 The BioInspired Institute, Syracuse University, Syracuse, NY 13244, USA

**Keywords:** cellulose, PEG, modification, hydrogels, water treatment

## Abstract

Pollution with organic dyes is one of the most typical environmental problems related to industrial wastewater. The removal of these dyes opens up new prospects for environmental remediation, but the design of sustainable and inexpensive systems for water purification is a fundamental challenge. This paper reports the synthesis of novel fortified hydrogels that can bind and remove organic dyes from aqueous solutions. These hydrophilic conetworks consist of chemically modified poly(ethylene glycol) (PEG-m) and multifunctional cellulose macromonomers (“cellu-mers”). Williamson etherification with 4-vinylbenzyl chloride (4-VBC) is used to modify PEGs of different molecular masses (1, 5, 6, and 10 kDa) and cellobiose, Sigmacell, or Technocell™ T-90 cellulose (products derived from natural renewable resources) with polymerizable/crosslinkable moieties. The networks are formed with good (75%) to excellent (96%) yields. They show good swelling and have good mechanical properties according to rheological tests. Scanning electron microscopy (SEM) reveals that cellulose fibers are visibly embedded into the inner hydrogel structure. The ability to bind and remove organic dyes, such as bromophenol blue (BPB), methylene blue (MB), and crystal violet (CV), from aqueous solutions hints at the potential of the new cellulosic hydrogels for environmental cleanup and clean water safeguarding.

## 1. Introduction

Water is the most valuable natural resource on the planet, but only about 1% of water reserves is available for household or industrial use. As water demand is expected to continue rising to support the growing population [1], humanity finds itself in need of efficient wastewater treatment systems [2]. This makes environmental remediation, or so-called environmental cleanup, one of the most challenging and important tasks of the 21st century. The removal of water pollutants from the environment should be carried out in a sustainable, eco-friendly, and cost-effective way [3]. Recently, various research groups have been investigating multiple approaches; some methods involve the latest advances in nanotechnology and use metal [4] or biogenic nanoparticles [5], while others use plants and their associated microbes [6]. The former have too many uncertainties and difficulties in engineering the particles for optimal performance, and the latter have many unknowns regarding the ecological implications of phytoremediation. Hydrogel-based materials, on the other hand, are a great system for removing various contaminants from water as they are easy to handle, suitable for large-scale application, and predisposed to regeneration as well as reuse; most importantly, they have tunable chemical and physical properties [7]. Hydrogels are physically or chemically crosslinked 3D networks that allow for the retaining of large quantities of solute and binding of pollutants that are present in the solution. Using sustainable materials to develop such systems is another essential aspect that must be considered. As the materials society is trying to shift its focus from petroleum-based products towards bioderived and renewable ones [8,9], carbohydrates are receiving more and more attention and are being successfully used in a broad range of applications [10,11], including hydrogels for environmental cleanup [12].

Among the most common wastewater pollutants are organic dyes and heavy metals [13]. To this day, hydrogels remain one the most effective systems for their adsorptive removal [14,15]. Composite hydrogels consisting of various hydrophilic polymers combined with hydrophobic inorganic fillers (clays [16] or metal nanoparticles [17]) or bioderived materials, such as chitosan [18], lignin [19], and cellulose fibers [20,21], show great promise for environmental remediation. In one such example, Li and coworkers synthesized a modified porous composite hydrogel capable of adsorbing cationic dyes [22].

Cellulose-based hydrogels have come to light recently and have been extensively studied as materials suitable for wastewater treatment [23]. The addition of cellulose fibers to a polymer matrix has been proven to improve the mechanical as well as thermal properties of the resulting materials [24] and enhance their adsorption capacity. Alammar et al. have developed a strategy based on cellulose acetate to prepare core–shell hydrogel spheres that would effectively remove neonicotinoids from aqueous environments [25]. In a similar fashion, Godiya et al. have synthesized a composite hydrogel based on carboxymethyl cellulose (CMC) and polyacrylamide (PAM) for wastewater remediation. Their material was able to bind Cu^II^, Pb^II^, and Cd^II^ from water [26]. Another group reported on magnetic superabsorbent hydrogel nanocomposites, where magnetic iron oxide nanoparticles were synthesized in situ and incorporated into CMC/poly(acrylic acid) hydrogel. Such networks were able to effectively absorb crystal violet (CV) from an aqueous solution [27].

Another example of a semi-interpenetrating conetwork has been previously published, where a bioderived constituent is combined with a synthetic polymer. Chemically modified Gellan Gum was copolymerized with styrene [28]. The synthesized material was able to swell in aqueous and organic media, had improved mechanical and thermal properties, and showed binding ability toward several organic dyes. Cheng and his team have developed high-flux poly(ethylene glycol)-based thin film composite nanofiltration membranes on poly(ethersulfone) supports. The interfacial polymerization of amino-functional poly(ethylene glycol) (PEG) and trimesoyl chloride led to the formation of a multifunctional hydrophilic membrane that can be used for wastewater treatment or water softening. The water permeability of these membranes can be optimized by varying the pore sizes, surface charges, and hydrophilicity [29].

**Scheme 1:**
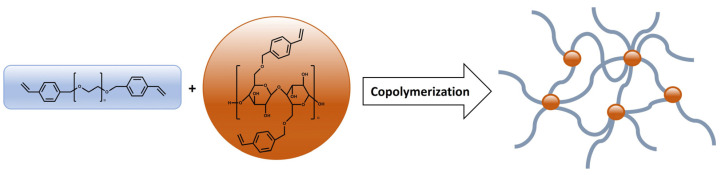
Crosslinking of styrene-modified PEG with multifunctional reactive “cellu-mer”.

Based on these previous results, the present study is aiming to create an environmentally safe system capable of swelling well in water and suitable for environmental remediation. The previously mentioned polymer—PEG—is chosen as it is soluble in water, has low intrinsic toxicity, and has been approved for internal consumption by the United States Food and Drug Administration (US FDA) [30]. PEG is utilized in a broad range of industries, including, but not limited to, pharmaceutical [31], chemical [32], cosmetic [33], and agricultural [34] enterprises. The most recently reported PEG-based hydrogels that are able to bind dyes use epichlorohydrin as a crosslinker [35]; however, exposure to epichlorohydrin is known to cause irritation to the eyes, respiratory tract, and skin [36]. In distinction, this paper reports the synthesis of novel hydrogels, where modified soft PEGs are reacted with polymerizable biocompatible and sustainable cellobiose (CB-m) molecules or cellulose fibers, CELL-m and T-90-m (“cellu-mers” [37]), to form cellulose-fortified conetworks, Scheme 1. The ability of these hydrogels to absorb and remove several organic dyes, such as bromophenol blue (BPB), methylene blue (MB), and crystal violet (CV), from aqueous solutions is investigated.

## 2. Results and Discussion

### 2.1. Synthesis and Characterization of Chain-End-Modified PEGs

**Scheme 2:**
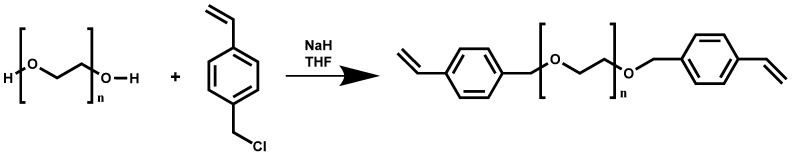
PEG chain-end modification with 4-vinylbenzyl chloride.

The modification of PEG chain ends with polymerizable groups is conducted via Williamson ether synthesis (Scheme 2).

**Figure 1:**
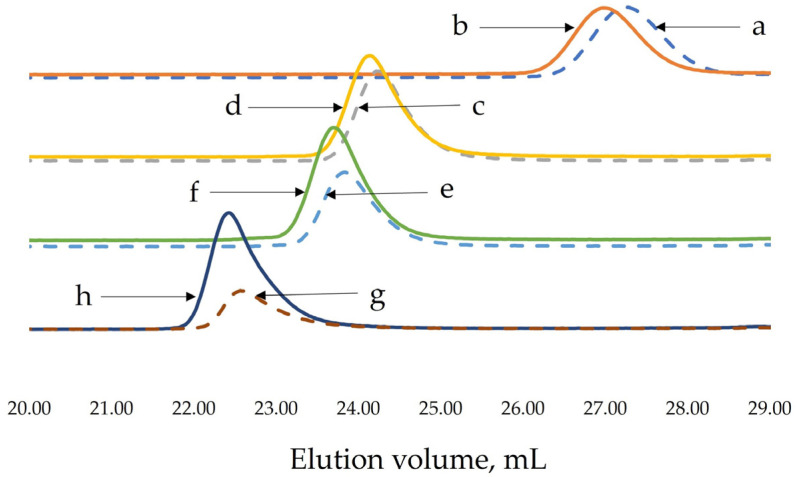
SEC chromatograms of initial PEGs (dashed line) and modified PEGs (solid line) in tetrahydrofuran (THF). (a) PEG 1 kDa; (b) PEG-m 1 kDa; (c) PEG 5 kDa; (d) PEG-m 5 kDa; (e) PEG 6 kDa; (f) PEG-m 6 kDa; (g) PEG 10 kDa; and (h) PEG-m 10 kDa.

The molecular masses of the synthesized reactive polymers are confirmed by size-exclusion chromatography (SEC). Figure 1 shows chromatograms of initial and functionalized PEGs. Modified PEGs, as expected, have peaks slightly shifted towards lower elution volumes, meaning that their hydrodynamic volumes (i.e., molecular masses) have increased. It is important to note that only one peak is visible for reaction products, indicating that both chain ends are quantitatively capped during the modification reaction. Table 1 contains the apparent molecular masses of the polymers, calculated from SEC analyses.

The degree of modification for PEG samples was studied using ultraviolet–visible (UV–Vis) spectroscopy (Figures S1 and S2). The degrees of modification for each of the PEG samples were as follows: 2.01 ± 0.28 for PEG-m 1 kDa, 2.02 ± 0.29 for PEG-m 5 kDa, and 1.75 ± 0.20 and 1.64 ± 0.29 for PEG-m 6 kDa and PEG-m 10 kDa, respectively. The lower degrees of modifications for higher-molecular-mass PEGs may be related to decreased solubility as the molecular mass increases. Raising the temperature during the reaction did not lead to any significant change in the degree of modification.

**Figure 2:**
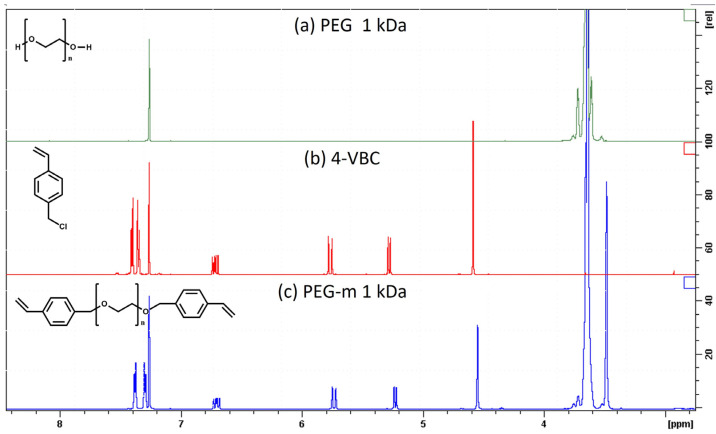
^1^H NMR spectra of (**a**) PEG 1k; (**b**) 4-VBC; and (**c**) PEG-m 1k. Analyses were performed in CDCl_3_.

Figure 2 shows the proton nuclear magnetic resonance spectroscopy (^1^H NMR) spectra of the starting compounds PEG 1k, 4-VBC, and the modified PEG-m 1k. This technique is able to reveal and confirm the expected structural changes in the synthesized molecules. PEG-m 1k contains signals from both PEG and vinyl benzyl fragments. As expected, peaks corresponding to the aromatic rings (7.2–7.5 ppm), double bonds (5–6.8 ppm), and benzyl CH_2_ groups (4.4–4.6 ppm) are slightly shifted in PEG-m compared to the 4-VBC, as groups in modified molecules have different chemical environments. Figure S3 compares all four modified PEG samples—1, 5, 6, and 10k. More detailed ^1^H and ^13^C NMR spectra of modified PEG-m 1k with assigned peaks are shown in Figures S4 and S5. Two-dimensional NMRs are shown in Figures S6 and S7.

### 2.2. Synthesis and Characterization of Hydrogels Based on PEG-m, Cellobiose, and Cellulose Crosslinks

The radical polymerization of modified PEGs and “cellu-mers” was conducted in THF using 2,2′-azobisisobutyronitrile (AIBN) as the initiator at 65 °C to crosslink the two components. Different weight ratios of crosslinker/PEG-m/AIBN were used, such as 0/99.5/0.5—no crosslinker, 99.5 wt% of PEG-m, and 0.5 wt% of AIBN in the system. Accordingly, 1/98.5/0.5 and 10/89.5/0.5 systems have one and ten weight percent of crosslinker, respectively. Synthesized networks were extracted with THF for three days to remove unreacted PEGs. Gel yields were calculated for each sample, and the swelling degree (SD) was measured and calculated following Equation (1) (see Materials and Methods, Section 3.2.5). It is worth mentioning that, in the absence of an initiator in the system, thermal crosslinking was also observed, and all of the results are presented in Tables S1–S3. Only gels with PEG-m 1, 5, and 6 kDa could be obtained with high yields using the described approach. The solubility of high-molecular-mass PEG chains in THF affected their reactivity, leading to low yields of PEG 10 kDa hydrogels.

Hydrogel swelling is an important characteristic, especially in view of its use as a wastewater treatment material. The swelling degree (SD) of synthesized hydrogels and gel yields is presented in Table 2. Swelling experiments were conducted on extracted gels. As expected, hydrogels prepared without any crosslinker had SDs higher than samples containing a crosslinker in both solvents. The number of crosslinks in a gel, or so-called crosslink density, strongly affects the physical properties and swelling of a gel [38,39,40]. In a densely crosslinked polymer network, polymer chains are partially entangled and less mobile, so the swelling decreases. This can also be seen in presented systems when gels that are formed with 10 wt% crosslinker swell in THF significantly less than those prepared with one percent. Additionally, the length of a polymer chain affects the swelling properties. The longer the polymer chain, the more space between the crosslink junctions to uptake solvent and swell; however, this ample space also allows for the entanglement of polymer chains within the system, which may restrict the swelling and not allow the gel to swell at its full capacity [41,42].

**Figure 3:**
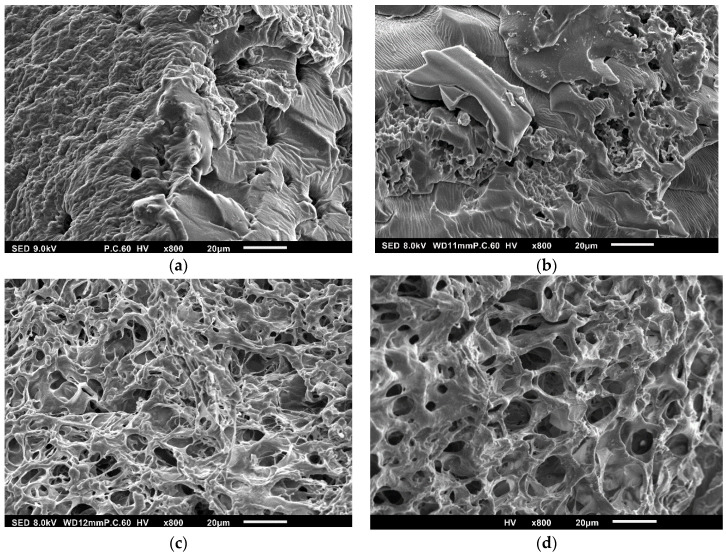
Scanning electron micrographs of synthesized hydrogels. (**a**) Self-crosslinked PEG-m 1k; (**b**) PEG-m 1k/1 wt% CB-m; (**c**) self-crosslinked PEG-m 6k; and (**d**) PEG-m 6k/1 wt% CB-m.

**Figure 4:**
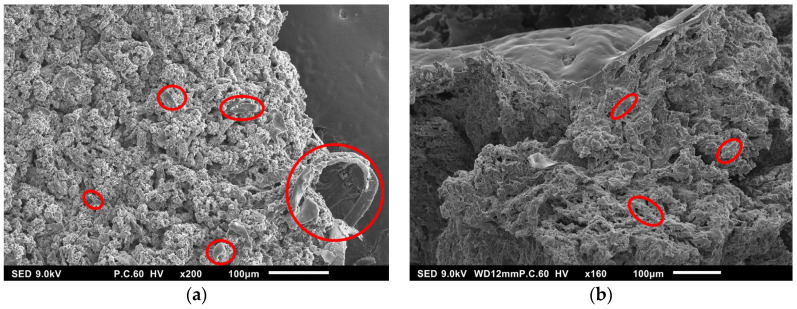
Scanning electron micrographs of hydrogels synthesized with 1 wt% CELL-m. (**a**) PEG-m 1k/CELL-m; (**b**) PEG-m 6k/CELL-m. Visible cellulose fibers are encircled in red.

The surface morphology of synthesized hydrogels was investigated via scanning electron microscopy (SEM). Samples were swollen in water and then cryofractured to reveal their inner structures. Hydrogels prepared with PEG-m 1k with no crosslinker (Figure 3a) and hydrogels prepared with PEG-m 1k/cellobiose-m (Figure 3b) have remarkably similar morphologies, implying that the addition of cellobiose-m does not substantially affect the segment arrangement of the resulting network. Gels prepared with PEG-m 6k and PEG-m 6k/cellobiose-m have interconnected porous structures and are also visually similar (Figure 3c,d). Micrographs of hydrogels prepared with PEG-m 1k and PEG-m 6k using 1 wt% cellulose Sigmacell (Figure 4a and Figure 4b, respectively) reveal that cellulose fibers (encircled in red) are embedded into the hydrogel network and are visible on the micrographs upon cryofracturing.

**Figure 5:**
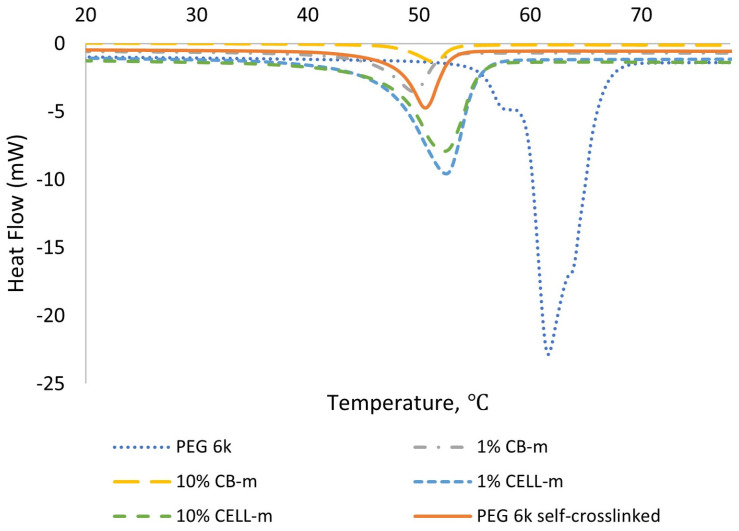
DSC traces of PEG-m 6 kDa hydrogels and starting material (PEG 6k).

The mobility of PEG segments within the network was investigated via differential scanning calorimetry (DSC). Melting transitions of hydrogels prepared with PEG-m 6k are shown in Figure 5. Typically, PEG chains in the crystal lattice are arranged as lamellae, with the chains in either extended or folded forms. For PEG 6k, there can be up to two folds in a crystal; however, the second one reportedly cannot be observed via DSC [43]. The unmodified PEG has seemingly three crystal fractions, the largest one having a melting temperature (T_m_) of 61.6 °C. The self-crosslinked PEG melts at 50.6 °C. Such results indicate that PEG chains, before modification, have more freedom to fold and form ordered crystals of different sizes that would require more energy to melt. On the other hand, PEG chains in the crosslinked sample are more restricted and undergo less chain folding (smaller crystals), so lamellae melt at lower temperatures. The measured degree of crystallinity (DC) is a good measure of the perfection of the crystals and their bulk content. PEGs are semicrystalline polymers, and depending on the sample preparation conditions and molecular mass, they may contain a portion of an amorphous fraction [44,45].

The degree of crystallinity (DC) was studied for the same PEG-m 6 kDa hydrogels (Table 3) and calculated via following Equation (2) (see Materials and Methods, Section 3.2.5). The results are in accordance with previous reports on thermal analyses of PEG 6k [46,47].

**Scheme 3:**
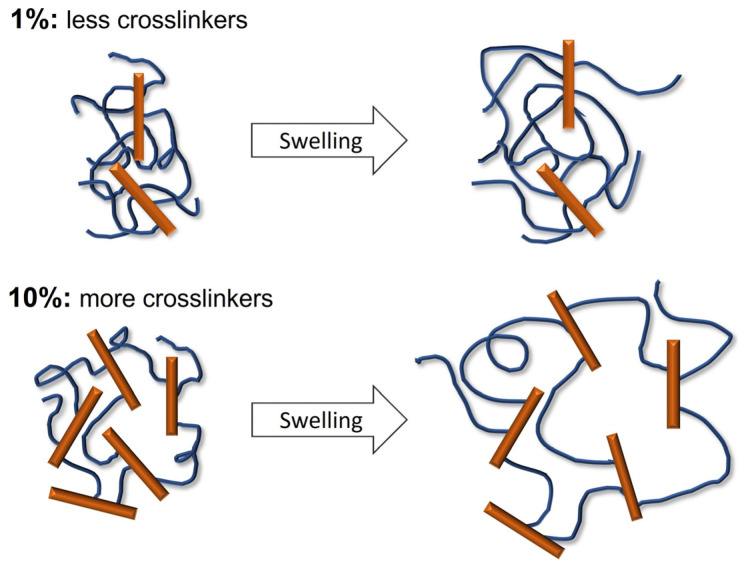
Hydrogel networks prepared with 1 and 10 wt% crosslinkers before and after swelling.

The amount and type of crosslinkers used can also affect the DSC results. In the data presented here, hydrogels prepared with 10 wt% CB-m melt at higher temperatures than those with 1 wt%, and hydrogels prepared with 1 and 10 wt% of added “cellu-mers” melt at the same temperature for both samples. The same trend is observed for other hydrogels prepared with CB-m and T90-m (Figures S8 and S9). This trend can be explained by taking into consideration the synthesis of hydrogels. Two concurrent processes occur during the polymerization reaction: the homopolymerization of PEG-m and PEG-m/crosslinker copolymerization. Both 1 and 10 wt% networks were synthesized with the same amount of initiator—0.5 wt%, meaning that the number of formed radicals initiating the reaction remains the same despite the increase in the number of crosslinkers added. These radicals are more scattered among the bundles of modified cellulose fibers, leading to a smaller amount of double bonds initiated on the surfaces of these fibers. Despite having a larger number of fortifying crosslinkers, the resulting hydrogels are possibly less densely crosslinked. This assumption is also supported by the DC, where it is markedly lower in CB gels (cellulose dimers) than with CELL gels (long cellulose chains), Table 3. The results of swelling experiments confirm this hypothesis (Table 2). Hydrogels prepared with 10 wt% of crosslinkers swell in water more than those prepared with 1 wt%, confirming the assumed lower crosslinking density. Scheme 3 is a schematic representation of two hydrogels prepared with 1 wt% and 10 wt% CELL-m before and after swelling. A DSC thermogram of cellulose microfibers has no sign of melting/crystallization transitions (Figure S10), and being immiscible with PEG should not interfere with the crystallization process.

**Figure 6:**
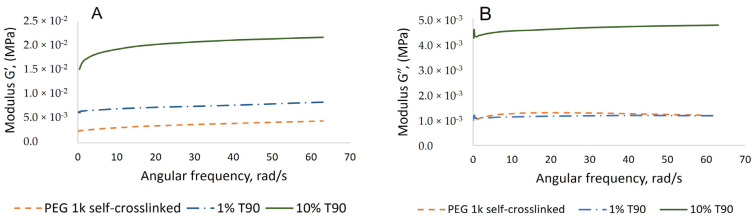
Frequency sweep of PEG-m 1k hydrogels, elastic (**A**) and viscous (**B**) moduli.

**Figure 7:**
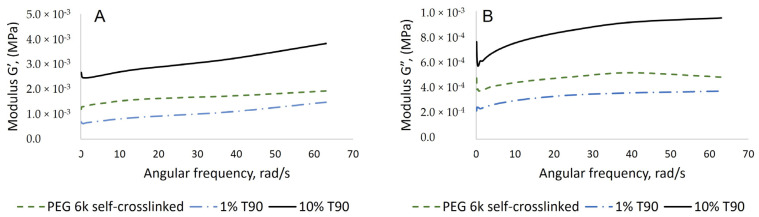
Frequency sweep of PEG-m 6k hydrogels, elastic (**A**) and viscous (**B**) moduli.

The structural integrity of a gel can be tested by standard rheological characterization. Properties such as “elastic modulus” (G′) and “viscous modulus” (G″) reveal the strength of a material [48]. Figure 6 and Figure 7 show the G’ and G” of hydrogels prepared with PEG 1k and 6k, respectively, crosslinked with modified cellulose T-90. Both G′ and G″ are strongly frequency independent, with G′ values larger than G″. These results indicate that all gels are “strong gels” that can store deformation energy over a broad frequency range due to their crosslinked structure [49,50]. Only Technocell-90-based hydrogels are shown here for simplicity, the rest of the data is presented on Figures S11–S14.

**Figure 8:**
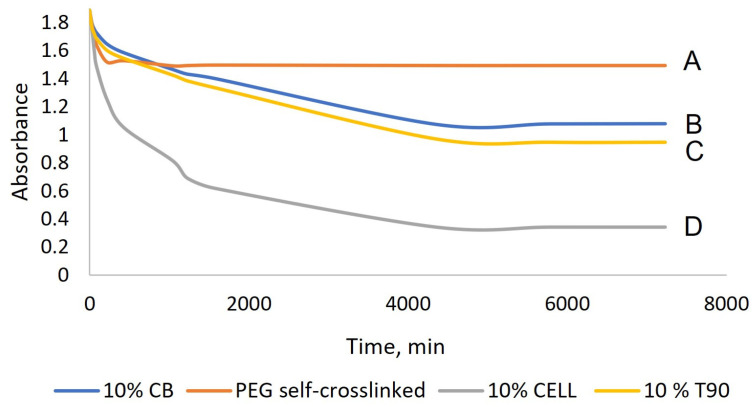
BPB encapsulation by PEG-m 6k hydrogels. (A) Self-crosslinked PEG-m; (B) 10% CB-m; (C) T90-m; and (D) 10% CELL-m. Absorbance was adjusted for the amount of hydrogel used.

The dye binding capability of hydrogels was tested using anionic (bromophenol blue, BPB) and cationic (crystal violet, CV; methylene blue, MB) dyes. They are among the most widely used dyes employed in the textile (cotton and silk) and paper (printing ink) industries [51,52], and could serve as good test substrates. Hydrogels based on PEG-m 6k with 10 wt% of various crosslinkers were used (Figure 8). These materials were chosen for two reasons: The PEG 6k network has a larger swelling degree and, according to SEM, its pores are interconnected, which promises better dye absorption [53,54]. In addition, it is expected that an increased amount of cellulose (10 wt% of crosslinker) may provide a more noticeable difference compared to the self-crosslinked PEG-m 6k. Despite the measured large swelling degree, a gel containing no crosslinkers showed no significant binding ability. It bound only 0.45 mg of BPB per 1 g of hydrogel and quickly reached equilibrium. Hydrogels crosslinked with CB-m and T90-m bound 2.75 mg/g and 3.1 mg/g, respectively. Gels prepared with 10 wt% of CELL-m showed the best binding capability among all of the measured samples and bound 5.22 mg/g of BPB. Since the CELL-based hydrogel showed the best BPB binding ability, it was used to analyze the adsorption capability of CV and MB.

**Figure 9:**
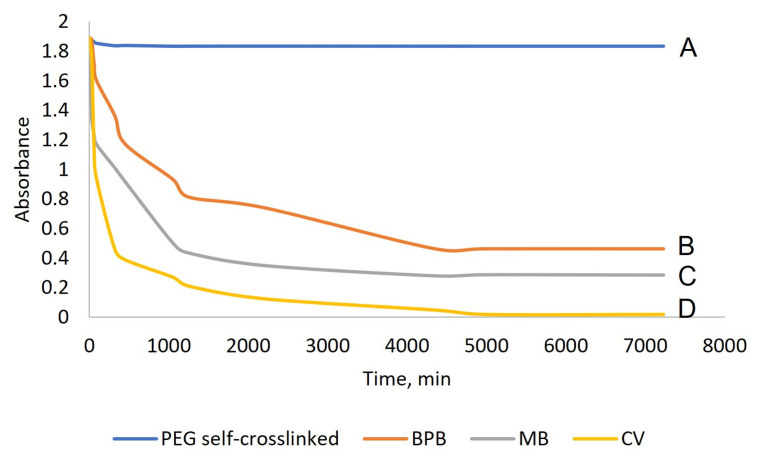
Comparison of organic dye adsorption by PEG-m 6k gels. (A) Self-crosslinked, BPB; (B) 10 w% CELL, BPB; (C) 10 w% CELL, MB; and (D) 10 w% CELL, CV. Absorbance was adjusted for the amount of hydrogel used.

An analysis reveals that the best absorption capability was in the case of CV (66.98 mg/g), followed by MB (8.5 mg/g) (Figure 9, Table 4). The CV absorption is notably better than other already-reported systems. For example, a CMC-based magnetic hydrogel binds 35 mg/g of CV at similar conditions [27] (p. 2). Other nanocomposite hydrogels could achieve only 28 mg/g of CV absorbed [55].

It is known that a material’s absorption capacity is determined not only by the surface and bulk characteristics of an absorbent but also by the properties and structures of absorbates [56]. Several factors can affect the binding propensity of a dye [57,58]: When dye molecules approach a hydrogel in a solute, they enter the network due to the osmotic pressure. If pores are too small, dye molecules cannot enter due to steric hindrance; however, the investigated gel is built of PEG-m 6k and the pores are sufficiently large to accommodate all three dyes. Thus, other binding forces must act to keep the absorbed species inside the network. Table 5 shows the chemical structures of each dye studied and pictures of gels before/after the corresponding dye absorptions. MB is the smallest molecule among those presented. If steric hindrance is the main factor that affects dye binding, MB, a cationic dye, would show the best result compared to CV, a cationic dye; however, the water solubility of MB is ~10 times higher than that of CV (43.6 g/L vs. 1 g/L, respectively) [59]. This means that the partition coefficient of MB will be substantially lower, since hydrophobic–hydrophobic interactions with the styrene-modified cellulose bundles will be much less pronounced. Indeed, a previous study has shown that MB partitions into poly(ethylene oxide)-*b*-poly(propylene oxide)-*b*-poly(ethylene oxide) much less than in pure poly(ethylene oxide) of 1 or 6 kDa [60].

## 3. Materials and Methods

### 3.1. Materials

Sigmacell cellulose (Type 101, Sigma-Aldrich, St. Louis, MO, USA) and Technocell™ cellulose fibers, Technocell 90 (T-90), Cellulose Filler Factory Corporation (Chestertown, MD, USA), were used as received. PEG 1 kDa, 6 kDa, and 10 kDa, 4-vinylbenzyl chloride (4-VBC, 90%), 2,2′-azobisisobutyronitrile (AIBN, 98%), and sodium hydride (NaH, dry, 95%) were supplied by Millipore Sigma (St. Louis, MO, USA). PEG 5 kDa was purchased from Polymer Source, Inc. (Dorval, QC H9P 2X8, Canada), and D-(+)-cellobiose was purchased from TCI America (Portland, OR, USA). Tetrahydrofuran (THF, EMD Millipore Corporation, Burlington, MA, USA), chloroform (J. T. Baker, Phillipsburg, NJ, USA), and methanol (Burdick & Jackson, Muskegon, MI, USA) as well as hexanes (Brookfield, CT, USA) were used as received.

### 3.2. Methods

#### 3.2.1. Preparation of Modified PEG (PEG-m)

Firstly, 1 g of a starting compound (PEG of a certain chain length) was dissolved in 3 mL of anhydrous THF. Then, 2.2 eq of 4-VBC was added. After stirring the mixture for 5 min, NaH was added into the reaction system and kept for 8 h. After that time, MeOH was added to quench the reaction. Gradient column was conducted to purify the product (starting with chloroform and switching to chloroform/methanol/hexanes, 4/1/1), the process was monitored by thin-layer chromatography (TLC). The modified PEG (PEG-m) was obtained as pale-yellow waxy solid. The yields were as follows: PEG-m 1 kDa—92%, PEG-m 5 kDa—95%, PEG-m 6 kDa—92%, and PEG-m 10 kDa—89%.

#### 3.2.2. Preparation of Reactive “Cellu-Mers” Based on Cellobiose and Cellulose Microfibers

Three types of “cellu-mers” were prepared from cellobiose (CB), cellulose Sigmacell-(CELL), and Technocell (T-90). Cellulose T-90 differs from Sigmacell in terms of the size of cellulose fibers, ranging from 20 μm (Sigmacell) to 90 μm (Technocell 90). The synthesis of “cellu-mers” has been reported previously [37]: Briefly, 0.2 g of cellobiose or cellulose microfibers was dispersed in 3 mL of anhydrous DMSO and then 4-vinylbenzyl chloride (8 eq) as well as NaH were added. The reaction system was kept at room temperature (RT) for 24 h. After that time, MeOH was added to quench the reaction. The reaction mixture was extracted with hexanes (for cellobiose) or vacuum-filtered and washed with ethanol (for cellulose). The modified microfibers were obtained as a yellow-colored liquid in the case of cellobiose and pale, yellow-colored solids in the case of cellulose. Modified cellulose fibers contained up to 91 mg/g of added methyl styrene fragment in 1 g of the cellulose (CELL-m and T-90-m), and modified cellobiose (CB-m) had 3.4 methyl styrene fragments added to its molecule.

#### 3.2.3. Synthesis of Hydrogels Using PEG-m with Different Chain Lengths and “Cellu-Mers”

Necessary amounts of PEG-m, crosslinker (CB-m, CELL-m, or T-90-m), and AIBN were dissolved in anhydrous THF. The ratio of ingredients varied and is described in Tables S1–S3. Polymerization was conducted at 65 °C under an argon atmosphere for 2–8 h, and, after this, the solidified reaction mixture was extracted with THF to remove unreacted PEG-m in addition to being further analyzed. The amount of extracted PEG (% extractables) was determined gravimetrically.

#### 3.2.4. Characterization of Modified PEGs

- Size-Exclusion Chromatography (SEC)

Polymers were analyzed using a system consisting of an M510 pump, U6K universal injector, 486 tunable absorbance detector (all from Waters Corporation, Milford, MA, USA), and 250 dual refractometer/viscometer detector (Viscotek/Malvern Corporation). The separation was achieved over a set of three 5 µm Styragel columns (HR 2, 3, and 5, Waters Corporation) and calibrated with 17 narrow dispersity poly(styrene) standards with molecular masses between 0.162 kDa and 956 kDa in THF.

- Nuclear Magnetic Resonance Spectroscopy (NMR)

^1^H NMR spectra were recorded using deuterated chloroform as a solvent at 22 °C with a Bruker AVANCE 600 MHz instrument (Bruker, Billerica, MA, USA). Tetramethylsilane was used as an internal standard at temperature, T = 300 K. ^1^H: number of scans, NS = 16, delay time, TD = 65,536; ^13^C: NS = 1024, TD = 65,536. For 2D NMRs COSY: NS = 4, TD = 2048; HSQC: NS = 4, TD = 1658.

PEG-m 1 kDa: ^1^H NMR (CDCl_3_):  δ 3.62 (2H, s, CH_2_), 4.55 (2H, s, CH_2_), 5.22 (1H, d, J = 10.74 Hz), 5.72 (1H, d, J = 17.52 Hz), 6.72 (1H, dd, J = 10.95 Hz, J’ = 10.94 Hz), 7.29 (2H, d, J = 7.44 Hz), and 7.37 (2H, d, J = 7.53 Hz). ^13^C NMR (CDCl_3_):  δ 70.57, 72.96, 113.73, 126.21, 127.94, 136.57, 136.99, and 137.91.

- Ultraviolet–Visible (UV–Vis) Spectroscopy

Samples were analyzed using an Agilent 8453 UV–visible spectrophotometer (Agilent Technologies, Santa Clara, CA, USA). A calibration curve was built using 4-VBC solutions at various concentrations in THF, and synthesized products were analyzed with pure THF as the blank solution.

#### 3.2.5. Characterization of PEG-m Hydrogels

- Swelling Studies

Swelling behavior (the ability of the gel to retain compatible liquids) was studied via a gravimetric method. Here, a dry sample with a known weight was immersed in water at room temperature (RT). The measurement was repeated three times for each gel sample.

The increase in weight of swollen gel was checked every 10 min during the first hour and then every 20 min until no further increase was noted, and the swelling degree (SD) was calculated using Equation (1):

(1) $$\mathrm{SD}\;(\%)=[(W_{s}-W_{0})/W_{0}]\;\times \;100,$$

where $W_{s}$ is the weight of the swollen gel at time t and $W_{0}$ is the weight of the dry gel.

- Scanning Electron Microscopy (SEM)

The surface morphology of swollen extracted hydrogels was examined by a JSM 5800LV scanning electron microscope (JEOL, Tokyo, Japan). Those swollen in water samples were cryofractured and carbon-coated under a vacuum before the electron micrographs were recorded.

- Differential Scanning Calorimetry (DSC)

A differential scanning calorimeter (DSC), a Q200, (TA Instruments, New Castle, DE, USA), was used to determine the crystallization and melting transitions. Samples were analyzed in a heating–cooling–heating cycle from −60 to 80 °C at a scanning rate of 10 °C/min using a dry nitrogen flow. The second heating cycle was used to study the melting transition. The degree of crystallinity (DC) was calculated using Equation (2):

(2) $$\mathrm{DC}\;(\%)=[({∆H}_{m}/{{∆H}_{m}}^{0}]\;\times \;100,$$

where ${∆H}_{m}$ is the measured melting enthalpy and ${{∆H}_{m}}^{0}$ is 196.8 J/g for PEG 6k analyzed at identical conditions [61].

- Rheology Measurements

Rheological experiments were carried out using a Discovery Hybrid Rheometer (DHR3) with temperature-controlled lower Peltier plate geometry 8 mm (TA Instruments, New Castle, DE, USA). Previously water-swollen samples had a thickness of 2.0 mm. Frequency sweep experiments were performed on the hydrogels at 25 °C in the range of 0.01 to 60 rad/s. Experiments were carried out in triplicate, and the calculated values are reported as an average value.

- Adsorption Capability, or Dye Binding

A sample of extracted dry gel (about 5 mg) was immersed in a 3 mL solution of a corresponding dye (0.04 mg/mL). The absorbance of the supernatant solution was monitored over time via the use of UV–Vis spectroscopy. The Bouguer–Beer–Lambert equation was used [62] to calculate the residual concentrations. When all of the dye from the solution was absorbed, the solution was discarded and a fresh portion of dye solution was added; its absorbance was monitored. This process was repeated until no change in the absorbance of the supernatant solution was noticed.

## 4. Conclusions

This paper describes the successful synthesis of novel PEG-based hydrogels fortified via cellulose macromonomers. Chemically modified cellobiose or cellulose fibers were used to crosslink modified PEG chains of various lengths (1, 5, 6, and 10 kDa). The morphology, degree of swelling, and viscoelastic as well as thermal properties of the hydrogels were investigated. Their ability to bind and remove organic dyes from aqueous solutions was used as proof of principle. Gels prepared with 10 wt% of modified cellulose Sigmacell and PEG-m 6k showed the best binding capabilities by effectively removing organic dyes from the solution, the highest adsorbing achieved with the cationic dye, CV—67 mg/g. Future efforts will be focused on the improvement of the binding and release capabilities of the hydrogels in order to reuse them over several remediation cycles. The results obtained showed that cellulosic additives yield strong gels with good swellability and suitable pore structures that could be used as absorbing floaters or membranes for environmental cleanup. Data in the public domain indicate that adsorption technologies and materials are therefore economically feasible [63]. Compared to reverse osmosis, nanofiltration, and other technologies for wastewater purification, methods based on adsorption are the most efficient in their exploitation characteristics (simple design, low installation price, minimal energy consumption, low maintenance, and small cost per unit of treated volume) [64]. According to the available economic reports, wastewater treatment equipment market size is expected to grow at a compound annual growth rate (CAGR) of 4.2% from 2022 to 2030 [65,66]. This forecasts revenue growth at global levels; therefore, it is expected that companies will start investing in the development of new efficient wastewater treatment materials and systems. In addition to this, hydrogels based on naturally derived materials are reported to bind and release other substances, such as phytomedicine [67], peptides, proteins, probiotics [68], and even blood clotting drugs [69]. The abovementioned factors greatly broaden the impact of the research findings reported in this paper.

## Figures and Tables

**Table 1:** Apparent molecular masses and dispersity indices (Ð) of PEGs and modified PEGs obtained by SEC in tetrahydrofuran (THF).

Sample	M_n_^(a)^	M_w_^(b)^	M_p_^(c)^	Ð ^(d)^
PEG 1k	1300	1400	1500	1.09
PEG 1k-m	1500	1600	1700	1.08
PEG 5k	6500	7000	7200	1.05
PEG 5k-m	6900	7100	7600	1.04
PEG 6k	8100	8400	8800	1.04
PEG 6k-m	8700	9100	9500	1.04
PEG 10k	11,600	12,200	13,400	1.05
PEG 10k-m	15,100	15,600	15,700	1.03

^(a)^ Number average molecular mass; ^(b)^ weight average molecular mass; ^(c)^ molecular mass at the peak apex; and ^(d)^ dispersity index—M_w_/M_n_.

**Table 2:** Hydrogels synthesized from PEG-m 1 and 6 kDa.

Hydrogel	Crosslinker, wt%	PEG-m, wt%	AIBN, wt%	SD in THF, %	SD in H_2_O, %	Extractables, %	Gel Yield, %
	No crosslinker						
1 kDa	0	99.5	0.5	837 ± 16	1236 ± 75	5.30	94.70
6 kDa	0	99.5	0.5	1048 ± 371	1682 ± 132	7.6	92.4
	CB-m						
1 kDa	1	98.5	0.5	427 ± 31	628 ± 89	17.65	82.35
	10	89.5	0.5	356 ± 17	871 ± 95	20.20	79.80
6 kDa	1	98.5	0.5	561 ± 41	655 ± 181	13.7	86.3
	10	100	0.5	237 ± 46	779 ± 30	11.1	88.9
	CELL-m						
1 kDa	1	98.5	0.5	338 ± 24	568 ± 74	24.40	75.60
	10	89.5	0.5	125 ± 9	296 ± 9	13.25	86.75
6 kDa	1	98.5	0.5	652 ± 185	738 ± 12	10.1	89.9
	10	89.5	0.5	387 ± 35	1102 ± 170	8.4	91.6
	T-90-m						
1 kDa	1	98.5	0.5	411 ± 50	683 ± 136	3.50	96.50
	10	89.5	0.5	144 ± 4	323 ± 48	4.70	95.30
6 kDa	1	98.5	0.5	467 ± 45	376 ± 27	7.9	92.1
	10	89.5	0.5	305 ± 97	948 ± 122	21.4	78.6

**Table 3:** Thermal properties of PEG samples derived from DSC measurements.

Sample	T_m_, °C ^(a)^	∆H_m_, J/g ^(b)^	DC, % ^(c)^
PEG 6k	61.6	179.0	90.9
PEG-m 6k self-crosslinked	50.6	106.7	54.2
1% CB	49.5	41.6	21.1
10% CB	51.5	14.6	7.4
1% CELL	52.4	85.6	43.5
10% CELL	52.4	73.4	37.3

^(a)^ Melting temperature; ^(b)^ phase transition enthalpy; and ^(c)^ calculated degree of crystallinity.

**Table 4:** Adsorption capability of various dyes via 10 wt% CELL PEG 6k gels.

Dye	Adsorption Capability, mg/g
BPB	5.3
MB	8.5
CV	~67.0

**Table 5:** Structures of organic dyes and pictures of hydrogels before/after corresponding dye adsorption.

Bromophenol Blue (BPB)	Crystal Violet (CV)	Methylene Blue (MB)
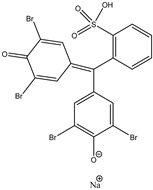	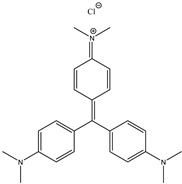	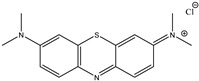
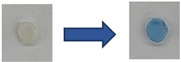	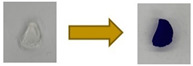	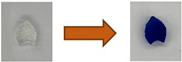

## Data Availability

All of the results obtained in this study are included in the paper and the supporting information. Any questions or requests can be referred to the corresponding author.

## Supplementary Materials

The following supporting information can be downloaded at: https://www.mdpi.com/article/10.3390/ijms24087558/s1.
