# Supplementary material for: Composite Hydrogels Based on Poly(Ethylene Glycol) and Cellulose Macromonomers as Fortified Materials for Environmental Cleanup and Clean Water Safeguarding
Source: Int J Mol Sci. 2023 Apr 20;24(8):7558. doi: 10.3390/ijms24087558 (PMC10144984; doi:10.3390/ijms24087558)
Supplement: Supplementary file 1 [file ijms-24-07558-s001.zip › ijms-2320384-supplementary.pdf]

Supporting Information

Composite Hydrogels Based on Poly(ethylene glycol) and Cellulose Macromonomers as Fortified Materials for Environmental Cleanup and Clean Water Safegarding

Dariya Getya [1,2], Alec Lucas [3] and Ivan Gitsov [1,2,4,*]

[1] Department of Chemistry, State University of New York—ESF, Syracuse, NY 132101, USA; dgetya@syr.edu
[2] The Michael M. Szwarc Polymer Research Institute, Syracuse, NY 13210, USA
[3] Department of Materials Science and Engineering, Purdue University, West Lafayette, IN 47907, USA; lucas120@purdue.edu
[4] The BioInspired Institute, Syracuse University, Syracuse, NY 13244, USA
[*] Correspondence: igivanov@syr.edu; Tel.: +1-315-470-6860

Table of contents:

Figure S1. UV-Vis analysis of modified PEGs. Page 2

Figure S2. Calibration curve that was used to calculate the degree of substitution of modified compounds. Page 2

Figure S3. Proton NMR spectra of a) PEG-m 10 kDa; b) PEG-m 6 kDa; c) PEG-m 5 kDa; d) PEG-m 1 kDa; e) 4-VBC. Page 3

Figure S4. ^{1}H NMR spectrum of PEG-m 1 kDa with peaks assigned. Page 3

Figure S5. ^{13}C NMR spectrum of PEG-m 1 kDa with peaks assigned. Page 4

Figure S6. COSY analysis of PEG-m 1 kDa with peaks assigned. Page 5

Figure S7. HSQC analysis of PEG-m 1 kDa with peaks assigned. Page 6

Figure S8. DSC traces of PEG 1 kDa hydrogels and starting material (PEG 1k). Page 6

Figure S9. DSC traces of PEG 5 kDa hydrogels and starting material (PEG 5k). Page 7

Figure 10. DSC thermogram of cellulose microfibers. Page 7

Figure S11. Frequency sweep of PEG-m 1k hydrogels, elastic modulus. Page 8

Figure S12. Frequency sweep of PEG-m 1k hydrogels, viscous modulus. Page 8

Figure S13. Frequency sweep of PEG-m 6k hydrogels, elastic modulus. Page 9

Figure S14. Frequency sweep of PEG-m 6k hydrogels, viscous modulus. Page 9

Table S1. Gels synthesized from PEG-m 1 kDa. Page 10

Table S2. Gels synthesized from PEG-m 5 kDa. Page 10

Table S3. Gels synthesized from PEG-m 6 kDa. Page 11

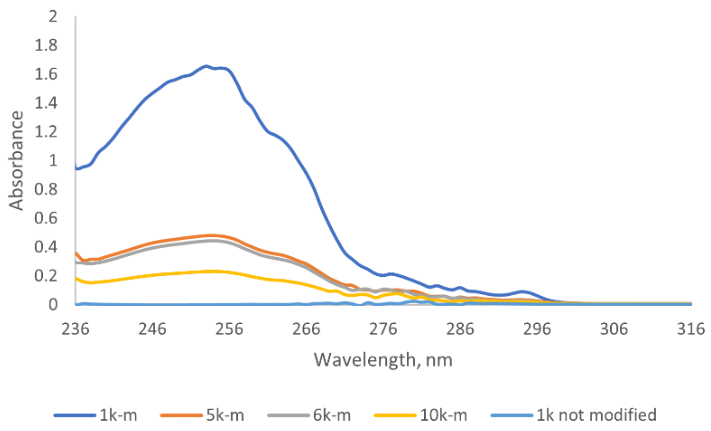

Figure S1. UV-Vis analysis of modified PEGs. The concentration of all samples is 3 mg/mL. Not modified PEG 1 kDa is shown too.

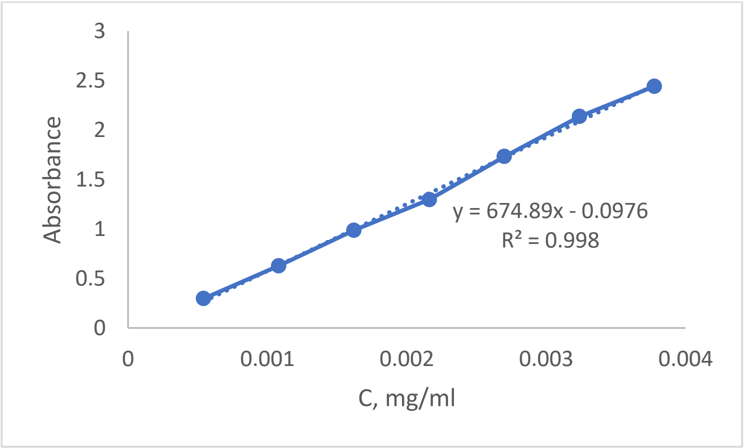

Figure S2. Calibration curve that was used to calculate the degree of substitution of modified compounds. UV-VIS absorbance of 4-VBC in THF at 254 nm.

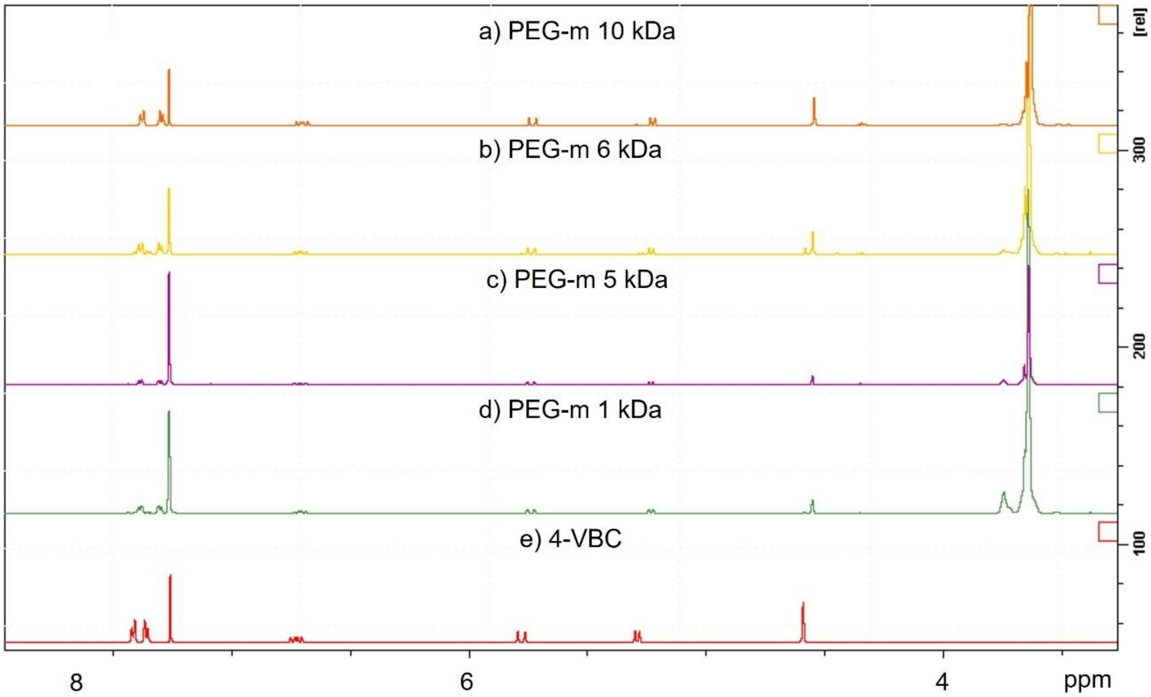

Figure S3. Proton NMR spectra of a) PEG-m 10 kDa; b) PEG-m 6 kDa; c) PEG-m 5 kDa; d) PEG-m 1 kDa; e) 4-VBC. Spectra recorded in deuterated chloroform.

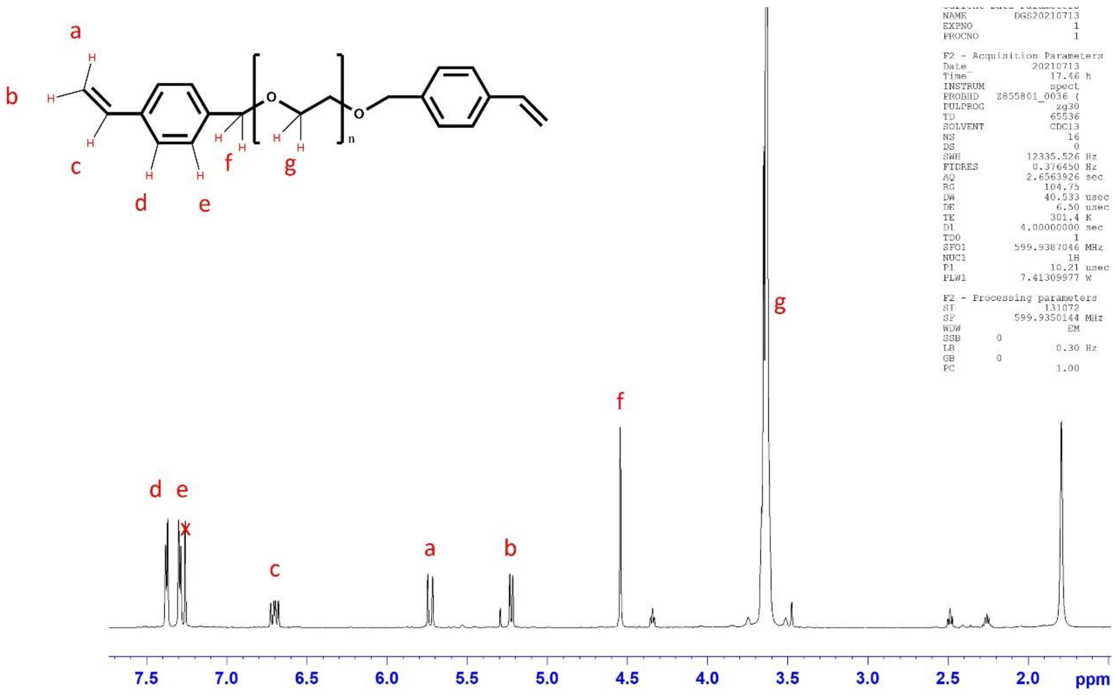

Figure S4. ^{1}H NMR spectrum of PEG-m 1 kDa with peaks assigned.

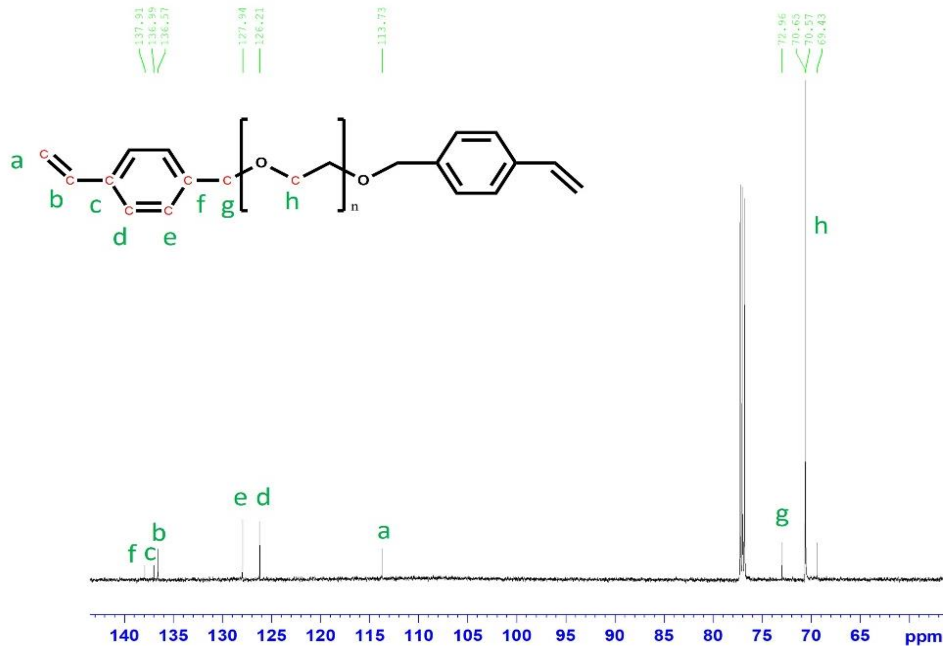

Figure S5. ^{13}C NMR spectrum of PEG-m 1 kDa with peaks assigned.

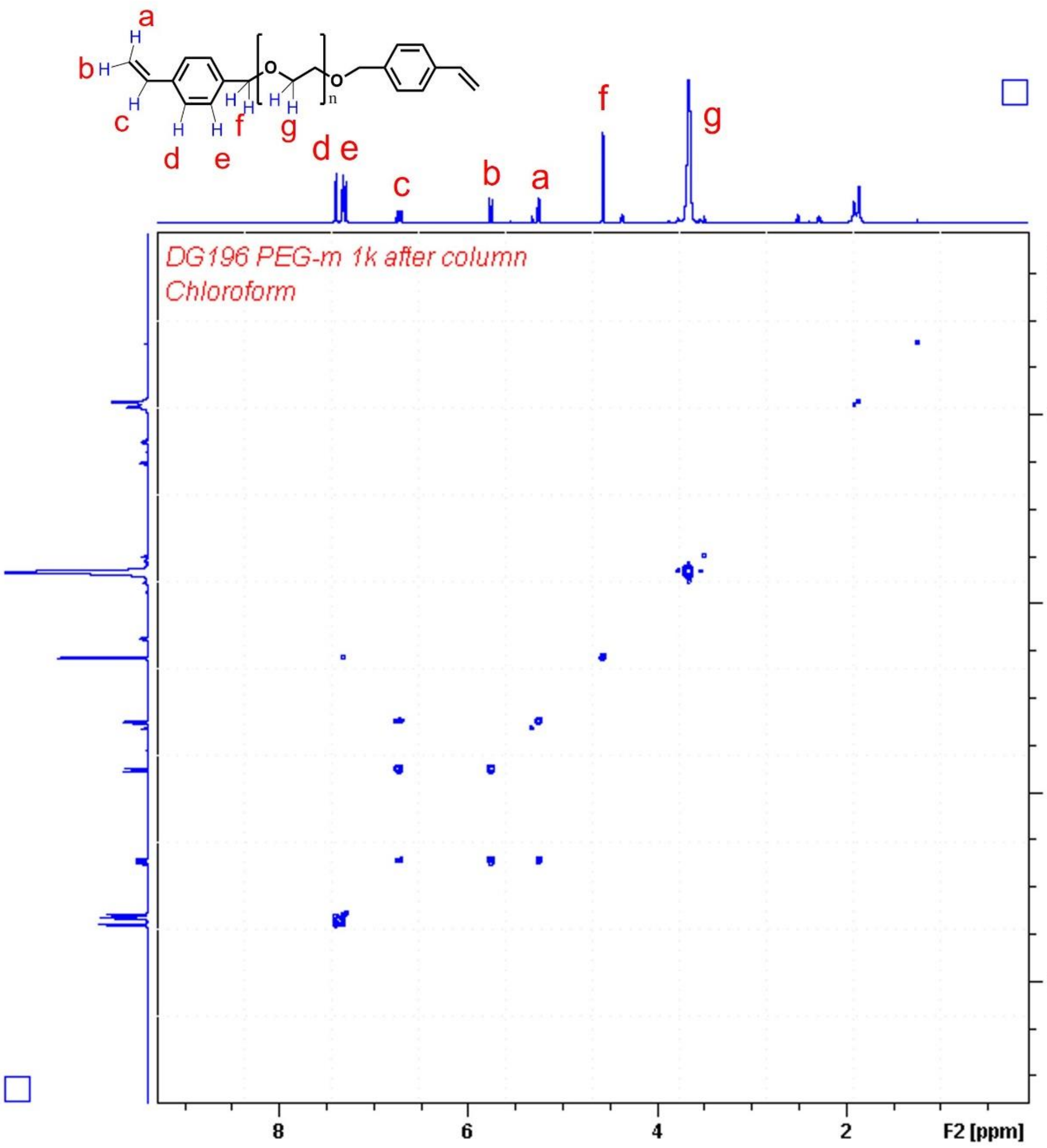

Figure S6. COSY analysis of PEG-m 1 kDa with peaks assigned.

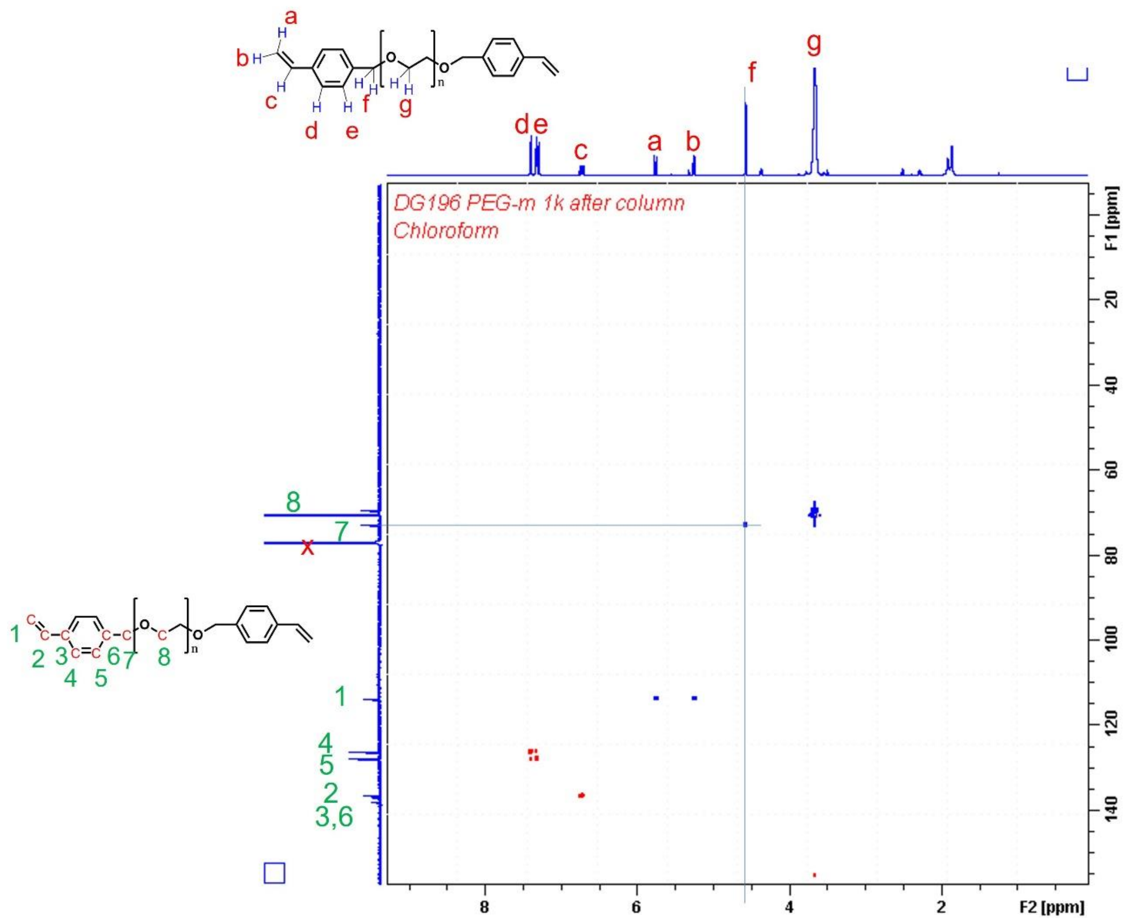

Figure S7. HSQC analysis of PEG-m 1 kDa with peaks assigned.

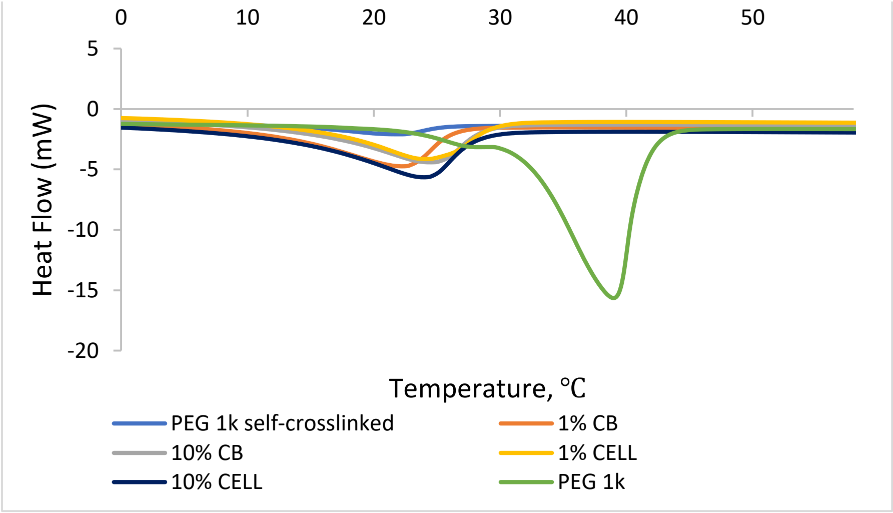

Figure S8. DSC traces of PEG 1k hydrogels and starting material (PEG 1k).

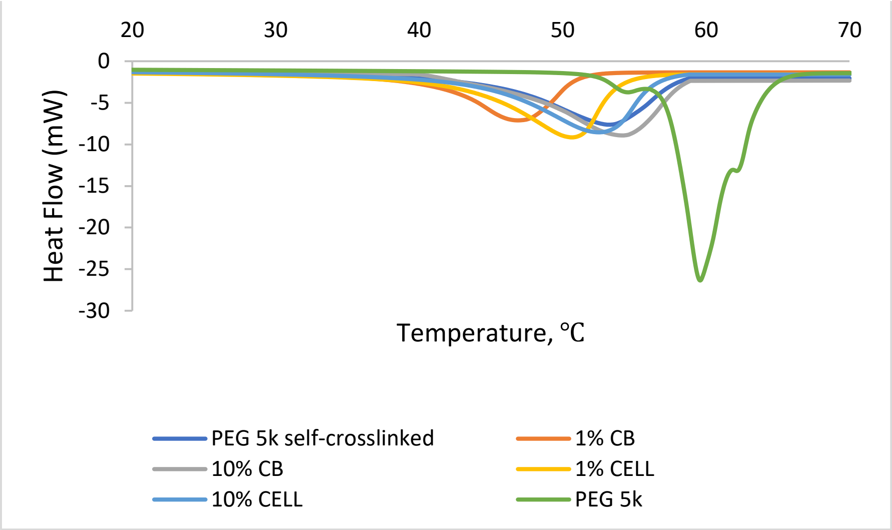

Figure S9. DSC traces of PEG 5k hydrogels and starting material (PEG 5k).

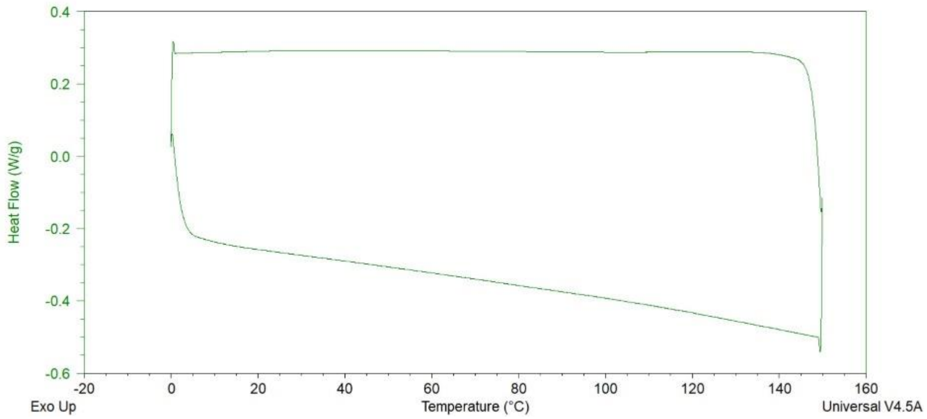

Figure S10. DSC thermogram of cellulose microfibers.

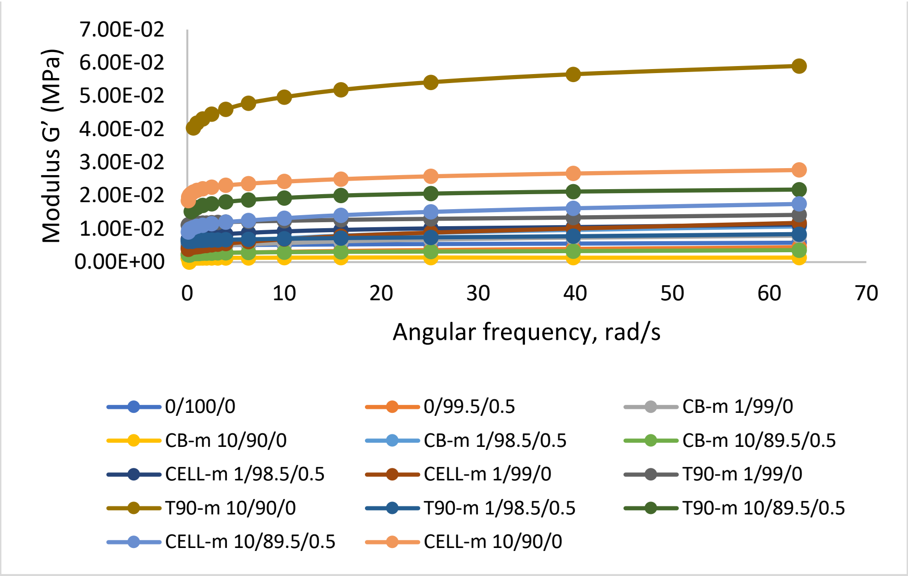

Figure S11. Frequency sweep of PEG-m 1k hydrogels, elastic modulus. The ratios of components are A/B/C, where A is a crosslinker, wt%; B is PEG-m, wt%; C is an initiator, wt%.

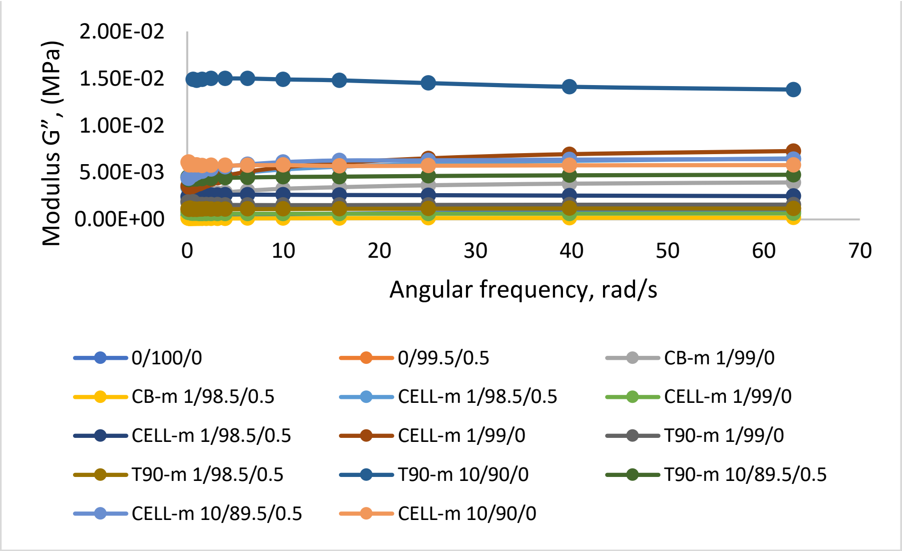

Figure S12. Frequency sweep of PEG-m 1k hydrogels, viscous modulus. The ratios of components are A/B/C, where A is a crosslinker, wt%; B is PEG-m, wt%; C is an initiator, wt%.

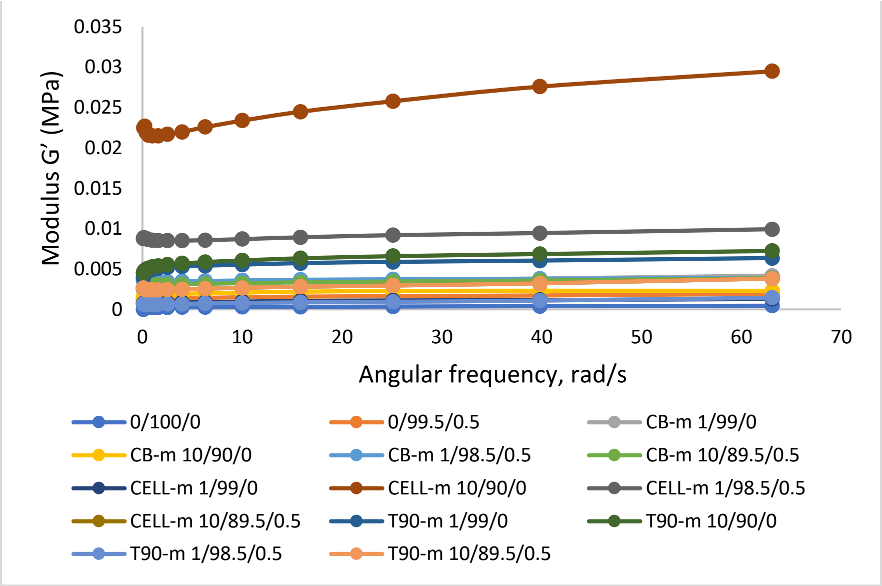

Figure S13. Frequency sweep of PEG-m 6k hydrogels, elastic modulus. The ratios of components are A/B/C, where A is a crosslinker, wt%; B is PEG-m, wt%; C is an initiator, wt%.

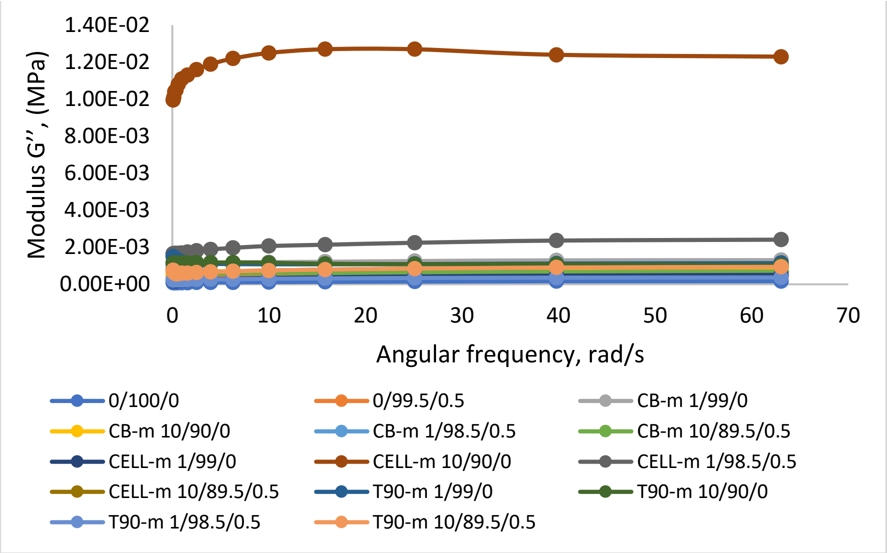

Figure S14. Frequency sweep of PEG-m 6k hydrogels, viscous modulus. The ratios of components are A/B/C, where A is a crosslinker, wt%; B is PEG-m, wt%; C is an initiator, wt%.

Table S1. Gels synthesized from PEG-m 1 kDa.

Crosslinker, wt%	PEG-m, wt%	AIBN, wt%	SD in THF, %	SD in H_2O, %	Extractables, %	Gel yield/ %
**No crosslinke**						
0	100	0	965±134	804±69	17.30	82.70
0	99.5	0.5	837±16	1236±75	5.30	94.70
**CB-m**						
1	99	0	305±38	488±66	30.65	69.35
10	90	0	276±28	573±96	25.05	74.95
1	98.5	0.5	427±31	628±89	17.65	82.35
10	89.5	0.5	356±17	871±95	20.20	79.80
**CELL-m**						
1	99	0	340±89	474±104	23.70	76.30
10	90	0	100±8	252±42	24.70	75.30
1	98.5	0.5	338±24	568±74	24.40	75.60
10	89.5	0.5	125±9	296±9	13.25	86.75
**T-90-m**						
1	99	0	297±21	518±33	4.60	95.60
10	90	0	89±26	349±116	4.40	95.40
1	98.5	0.5	411±50	683±136	3.50	96.50
10	89.5	0.5	144±4	323±48	4.70	95.30

Table S2. Gels synthesized from PEG-m 5 kDa.

Crosslinker, wt%	PEG-m, wt%	AIBN, wt%	SD in THF, %	SD in H_2O, %	Extractables, %	Gel yield/ %
**No crosslinker**						
0	100	0	985±252	1001±125	15.1	84.9
0	99.5	0.5	1005±187	1456±132	17.3	82.70
**CB-m**						
1	98.5	0.5	489±56	685±112	13.3	86.7
10	89.5	0.5	358±75	758±54	12.4	87.6
**CELL-m**						
1	98.5	0.5	652±185	789±65	12.7	87.3
10	89.5	0.5	458±85	1102±170	12.6	87.4
**T-90-m**						
1	98.5	0.5	652±185	738±12	13.6	86.4
10	89.5	0.5	281±35	1102±170	13.4	86.6

Table S3. Gels synthesized from PEG-m 6 kDa.

Crosslinker, wt%	PEG-m, wt%	AIBN, wt%	SD in THF, %	SD in H_2O, %	Extractables, %	Gel yield/ %
**No crosslinker**						
0	100	0	1113±312	1452±118	13.5	86.5
0	99.5	0.5	1048±371	1682±132	7.6	92.4
**CB-m**						
1	99	0	445±41	689±56	18.1	81.9
10	90	0	255±16	715±35	20.5	79.5
1	98.5	0.5	561±41	655±181	13.7	86.3
10	89.5	0.5	237±46	779±30	11.1	88.9
**CELL-m**						
1	99	0	463±66	576±154	12.9	87.1
10	90	0	281±154	939±153	21.5	78.5
1	98.5	0.5	652±185	738±12	10.1	89.9
10	89.5	0.5	387±35	1102±170	8.4	91.6
**T-90-m**						
1	99	0	370±49	548±56	20.9	79.1
10	90	0	248±66	643±146	25.0	75.0
1	98.5	0.5	467±45	376±27	7.9	92.1
10	89.5	0.5	305±97	948±122	21.4	78.6
